# Gene Therapy of *c-myc* Suppressor FUSE-Binding Protein-Interacting Repressor by Sendai Virus Delivery Prevents Tracheal Stenosis

**DOI:** 10.1371/journal.pone.0116279

**Published:** 2015-01-08

**Authors:** Daisuke Mizokami, Koji Araki, Nobuaki Tanaka, Hiroshi Suzuki, Masayuki Tomifuji, Taku Yamashita, Yasuji Ueda, Hideaki Shimada, Kazuyuki Matsushita, Akihiro Shiotani

**Affiliations:** 1 Department of Otolaryngology, Head & Neck Surgery, National Defense Medical College, Tokorozawa, Saitama, Japan; 2 DNAVEC Corporation, Tsukuba, Ibaraki, Japan; 3 Department of Surgery, Toho University School of Medicine, Ota-Ku, Tokyo, Japan; 4 Department of Molecular Diagnosis (F8), Chiba University Graduate School of Medicine, Chiba City, Chiba, Japan; Wayne State University, UNITED STATES

## Abstract

Acquired tracheal stenosis remains a challenging problem for otolaryngologists. The objective of this study was to determine whether the Sendai virus (SeV)-mediated *c-myc* suppressor, a far upstream element (FUSE)-binding protein (FBP)-interacting repressor (FIR), modulates wound healing of the airway mucosa, and whether it prevents tracheal stenosis in an animal model of induced mucosal injury. A fusion gene-deleted, non-transmissible SeV vector encoding FIR (FIR-SeV/ΔF) was prepared. Rats with scraped airway mucosae were administered FIR-SeV/ΔF through the tracheostoma. The pathological changes in the airway mucosa and in the tracheal lumen were assessed five days after scraping. Untreated animals showed hyperplasia of the airway epithelium and a thickened submucosal layer with extensive fibrosis, angiogenesis, and collagen deposition causing lumen stenosis. By contrast, the administration of FIR-SeV/ΔF decreased the degree of tracheal stenosis (*P* < 0.05) and improved the survival rate (*P* < 0.05). Immunohistochemical staining showed that c-Myc expression was downregulated in the tracheal basal cells of the FIR-SeV/ΔF-treated animals, suggesting that *c-myc* was suppressed by FIR-SeV/ΔF in the regenerating airway epithelium of the injured tracheal mucosa. The airway-targeted gene therapy of the *c-myc* suppressor FIR, using a recombinant SeV vector, prevented tracheal stenosis in a rat model of airway mucosal injury.

## Introduction

Successful methods for the treatment of tracheal stenosis are yet to be developed. Tracheal stenosis occurs in response to an injury of the airway mucosa, caused by prolonged endotracheal intubation, [1, 2] long-term tracheostomy, or an airway burn. A prolonged tracheostomy and repeated surgeries are needed in most cases, but they will significantly impair the patient’s ability to speak and swallow. Although various surgical techniques have been developed, such as balloon dilation, endoscopic laser resection, and end-to-end anastomosis, [3–5] the patient’s outcome can frequently be complicated by recurrent stenosis, due to the formation of new scars and to granulation following surgery, [5] as the surgery itself causes an injury to the airway mucosa. Novel approaches against post-surgical restenosis have long been needed. The modulation of wound healing in the airway mucosa represents a potential treatment option.

The sendai virus (SeV) has a strong affinity to the airway epithelium.[6, 7] The wild-type form of SeV causes respiratory tract infections in rodents, but is not pathogenic in humans. Its therapeutic use is predicted to be safer than that of DNA viral vectors, because the RNA genome of SeV does not go through a DNA phase. Therefore, the SeV vector has been suggested as a potential gene-transfer vector for the treatment of airway diseases.[8, 9] A fusion gene-deleted, non-transmissible Sendai virus vector (SeV/ΔF) was prepared in a preclinical study.[9] Its preventive effect in a model of tracheal obliteration following transplantation was reported without any systemic side effects.[10] We recently presented a novel model of acute mucosal injury, in which the tracheal mucosa of rats was scraped with a nylon brush through the tracheostoma, and we reported the successful gene transfer into the injured mucosa of those rats, through SeV/ΔF delivery.[9] In this model, the wound-healing response after the mechanical injury to the airway mucosa is seen as inflammation or fibrous proliferation in the regenerating tracheal mucosa.

*c-myc* is a regulator gene responsible for the upregulation of many genes that promote cell proliferation.[11] The elevated expression of c-Myc has been detected not only in a broad range of human cancers, [11] but also in chronic wounds.[12] The main cause of tracheal stenosis is the excessive cell proliferation that occurs within the limited tracheal space during wound healing. Therefore, the suppression of c-Myc expression represents a potential strategy for the treatment of tracheal stenosis.

The far upstream element (FUSE)-binding protein (FBP)-interacting repressor (FIR) is a *c-myc* transcriptional repressor.[13–15] FIR strongly suppresses *c-myc* transcription through its inhibition of the TFIIH/P89/XPB helicase (P89) activity.[14, 15] FIR also induces apoptosis by suppressing *c-myc*.[16]

The SeV-encoding FIR (FIR-SeV/ΔF) was identified as a candidate for cancer gene therapy.[17, 18] We reasoned that FIR-SeV/ΔF might also suppress *c-myc* in the tracheal mucosa, and thus show therapeutic potential in the treatment of tracheal stenosis. Therefore, we hypothesized that gene therapy using FIR-SeV/ΔF could prevent tracheal stenosis in an animal model of induced mucosal injury, via the inhibition of c-Myc caused by the expression of transduced FIR. The aim of this study was to provide evidence to support this hypothesis, using the above-mentioned rat model.

## Materials and Methods

### Animals

All protocols for the handling and the experimental use of animals were approved by the Committee on the Ethics of Animal Experiments and by the Safety Board on Recombinant DNA experiments of the National Defense Medical College (Permit Number: 14069 and 2012–24). All surgery was performed under anesthesia, and all efforts were made to minimize suffering. Twenty-eight adult, female Sprague–Dawley rats (200–220 g) were used.

### Preparation of non-transmissible recombinant SeV vectors

A fusion gene-deleted, non-transmissible SeV vector encoding FIR (FIR-SeV/ΔF; 8.2 × 10^9^ cell infectious units [CIU]/mL) was prepared as previously described [17]. FIR-SeV/ΔF was manufactured and provided by the DNAVEC Corporation (Tsukuba, Japan), and stored at -80°C until used.

### Surgical procedure

The animals were anesthetized with medetomidine (0.8 mg/kg; intraperitoneal (IP) injection) and with ketamine HCl (40 mg/kg; IP injection) during the surgery. The tracheostomy was performed at the tracheal rings 5 and 6. The laryngotracheal mucosa above the tracheostoma was scraped 10 times with a 1.8-mm nylon brush (Kobayashi Pharmaceutical, Osaka, Japan), through the tracheostoma as previously described.[9] To assess the preventive effect of FIR-SeV/ΔF, 19 rats were assigned to an untreated control group (*n* = 10) or to a FIR-SeV/ΔF-treated group (*n* = 9), respectively, at random. To confirm the transgene expression of FIR in the tracheal tissue using RT-PCR, nine rats were assigned to a normal group (*n* = 3), to an untreated control group (*n* = 3) or to a FIR-SeV/ΔF-treated group (*n* = 3), respectively, at random.

Ten microliters of FIR-SeV/ΔF (8.2 × 10^9^ CIU/mL) or of normal saline solution was administered through the tracheostoma to the injured airway mucosa, for three consecutive days after the tracheal injury. Animals were observed carefully following the surgery, to prevent an acute airway obstruction. The crusts that were attached to the tracheostoma were removed every 24 h until the animals were euthanized. Animals were euthanized with an intraperitoneal injection of a massive dose (150 mg/kg) of pentobarbital, and the tracheal tissue at the tracheal rings 1 and 2 was excised five days following the tracheal scraping. If the animals exhibited severe stridor, difficulties with ambulation, or a body weight loss that was greater than 20% due to tracheal stenosis, they were euthanized for humane reasons. The survival curves were drawn, and the survival rates were compared between the groups.

### Histological and quantitative analysis of lumen stenosis

To assess the pathological changes occurring at the airway mucosa, and to assess the degree of stenosis in the tracheal lumen, the excised tracheal tissues were fixed in 10% formalin, embedded in paraffin, and then cut into 4-μm-thick axial sections. The images of the H&E-stained axial sections were captured at a low-power magnification. The image J software, version 1.44p (National Institutes of Health, Bethesda, MD), was used to measure the area of the lumen-tracing mucosal surface as the narrowed lumen, and that of the cartilage surface as the initial lumen. The percentage of stenosis was calculated using the following formula, as was previously described [9]: (1 − area of the mucosal surface lumen/area of the tracheal cartilage lumen) × 100.

### Quantitative RT-PCR analysis of FIR RNA expression

To confirm the transgene expression of *FIR* in the tracheal tissue using quantitative real time RT-PCR, the tracheal tissues excised from the normal, the untreated control, and the FIR-SeV/ΔF-treated animals (*n* = 3 for each group) were prepared, as mentioned above. The tissue samples were fragmented into small pieces (<25 mg), and immersed into an RNAlater solution (Applied Biosystems, Tokyo, Japan). The RNA was isolated using an RNeasy Mini Kit (Qiagen, Valencia, CA). The primer sequences that were used are as follows: human FIR forward (exon 2) 5′-CCATAGCTCTCCAGGTCA-3′ and reverse (exon 6) 5′-CGTAGACGCGGCACATGA-3′; rat β-actin forward 5′-TGGAGAAGAGCTATGAGCTGCCTG-3′ and reverse 5′-GTGCCACCAGACAGCACTGTGTTG-3′.[19, 20] The mRNA levels of the relevant molecules were measured using quantitative real-time RT-PCR, with the One Step SYBR PrimeScript RT-PCR Kit (Takara Bio, Shiga, Japan) in the Thermal Cycler Dice Real Time System II (Takara Bio, Shiga, Japan). All reactions were performed in triplicates using a PCR assay, and under the same cycling conditions: The reverse transcription reaction was maintained at 42°C for 5 min, and was ended with an incubation step at 95°C for 10 s. The PCR step was carried out for 40 cycles at 95°C for 5 s; 60°C for 30 s; the final dissociation step was carried out at 95°C for 15 s, 60°C for 30 s, and 95°C for 15 s. The levels of accumulated fluorescence were analyzed using the second derivative maximum method, and the ΔΔCt method with the LightCycler data analysis software, TP 900 version 4.02 (Takara Bio, Shiga, Japan), following the melting-curve analysis, [21] and then the expression levels of the target genes were normalized to the expression level of β-actin in each sample. The results are shown as the mean ± standard error of the mean (SEM) of the three independent measurements.

### Immunohistochemistry for confirmation of the suppression *c-Myc* expression

Immunohistochemistry was used to confirm that the SeV-encoded FIR suppressed c-Myc expression. Following the deparaffinization and the hydration of the tracheal specimens from the untreated control and the FIR-treated animals, the slides were covered with 10 mM of sodium citrate buffer, pH 6.0, and were heated in an autoclave for antigen unmasking at 120°C for 5 min. The endogenous peroxidase activity was blocked using 3% H_2_O_2_ in methanol for 5 min. The streptavidin-biotin complex method (VECTASTAIN Elite ABC mouse IgG Kit PK-6102; Vector Laboratories, Inc., Burlingame, CA) was used according to the manufacturer’s instructions. The slides were incubated for 1 h with an anti c-Myc primary antibody (9E10, sc-40; Santa Cruz Biotechnology, Inc., Dallas, Texas) at room temperature in a moisture chamber. The slides were then incubated with a secondary biotinylated antibody solution and with the VECTASTAIN ABC Reagent at room temperature, each for 30 min, and then visualized with 3,3′-diaminobenzidine (DAB) and counterstained with hematoxylin. The c-Myc expression was observed under high-magnification microscopy, and the images were captured using a CCD camera (DP26; Olympus, Tokyo, Japan). To quantify the c-Myc-positive area in the nucleus, the regions of interest (ROIs) in a given display image at a 400-fold magnification were carefully defined over the mucosa of the trachea. Three ROIs were randomly cropped in each captured image. To quantify the c-Myc-positive area in the nucleus, the percentage of DAB-stained area in the ROIs was calculated using the following formula: (DAB-positive area/hematoxylin-positive area) × 100, using the Image J software, version 1.44p (National Institutes of Health).

### Statistical analysis

The results are shown as the mean ± standard error of the mean (SEM). To evaluate the significance of the differences in the percentages of stenosis, in the average mRNA expression levels, and in the DAB-positive areas between the two groups, Mann-Whitney U-tests were performed. The analysis of survival was performed using the log-rank test with the JMP software, version 10.0.0 (SAS Institute Inc., USA). The significance was determined using a *P*-value < 0.05.

## Results

### FIR-SeV/ΔF prevents tracheal stenosis

**Figure 1:**
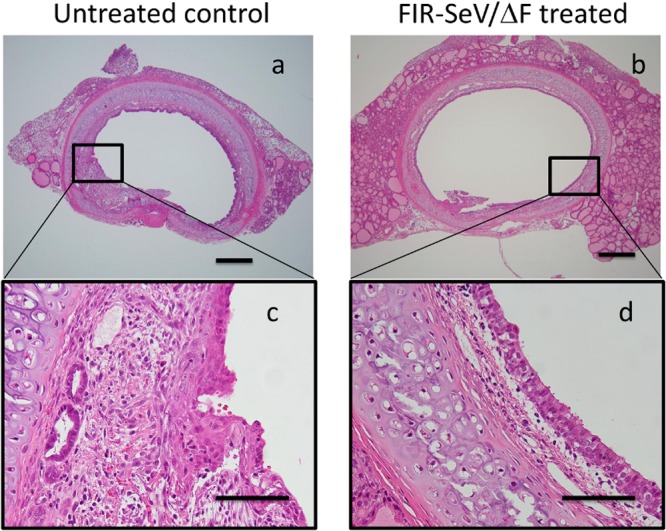
Representative H&E-stained axial sections of the rat trachea five days following the scraping of the tracheal mucosa. The untreated controls (tracheal scraping only (n = 10)), (**a, c**) showed both hyperplasia of the airway epithelium and a thickened submucosal layer with extensive fibrosis, angiogenesis, and collagen deposition causing lumen stenosis. The FIR-SeV/ΔF-treated animals, in which FIR-SeV/ΔF was administered into the tracheal mucosa following tracheal scraping (n = 9), (**b, d**), showed a reduction in the extent of hyperplasia of the tracheal epithelium. The scale bar in (**a, b**) and (**c, d**) indicates 500 μm and 100 μm, respectively.

**Figure 2:**
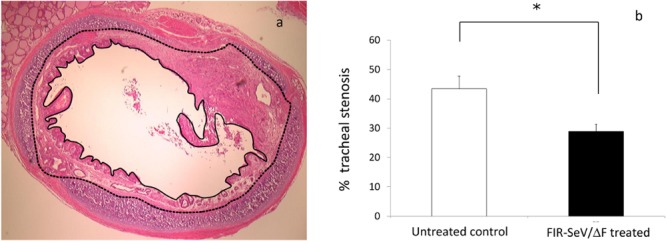
Quantitative analysis of tracheal stenosis. The percentage of stenosis five days following tracheal scraping was calculated using the following formula: (1 − the area of the mucosal surface lumen (solid line)/the area of the tracheal cartilage lumen (dotted line)) × 100 (**a**). The animals in which FIR-SeV/ΔF was administered (black) displayed a significantly lower percentage of stenosis than the untreated controls (white) (**b**). The results are expressed as the mean + SEM (bars). *P < 0.05 using the Mann–Whitney U test.

To assess the preventive effect of FIR-SeV/ΔF in a rat model of induced mucosal injury, we evaluated the pathological changes in the tracheal lumen and the degree of lumen stenosis. Rats that had their airway mucosa scraped were administered a normal saline solution (untreated controls) or FIR-SeV/ΔF through the tracheostoma. The pathological changes in the tracheal lumen were assessed five days after following. Representative hematoxylin–eosin (H&E)-stained axial sections of the trachea of untreated controls (Fig. 1a,c) revealed hyperplasia of the airway epithelium, and a thickened submucosal layer with extensive fibrosis, angiogenesis, and collagen deposition causing lumen stenosis. By contrast, the FIR-SeV/ΔF-treated animals (Fig. 1b,d) showed a decrease in the wound-healing response in the airway mucosa, when compared to the untreated controls. Hence **F**IR-SeV/ΔF decreased the degree of tracheal stenosis (28.9 ± 7.30% vs. 43.4 ± 12.4%, respectively; *P* < 0.05) (Fig. 2a,b), and thus prevented tracheal stenosis following a mucosal injury.

### FIR-SeV/ΔF improves the survival rate

**Figure 3:**
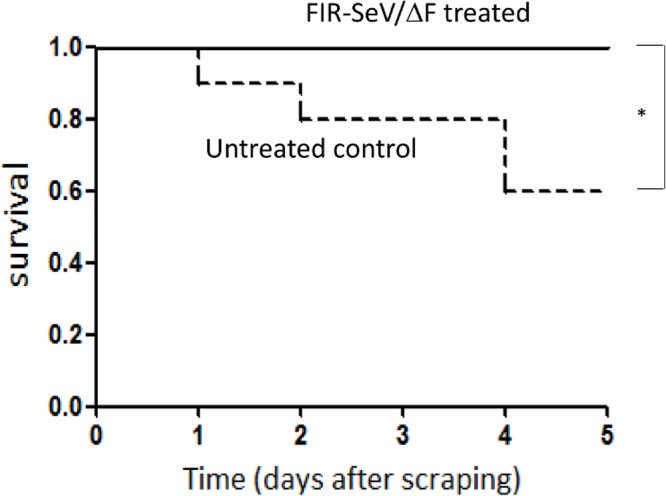
Kaplan–Meier survival curves of rats with tracheal stenosis. At the start of the study, 10 untreated rats and nine FIR-SeV/ΔF-treated rats were included. A survival analysis was performed using the log-rank test. The FIR-SeV/ΔF-treated animals (solid line) displayed a significantly higher survival rate than the untreated control animals (dotted line) five days following the scraping of the tracheal mucosa. *P < 0.05 using the log-rank test.

Animals were euthanized five days following tracheal scraping, or when the animals displayed severe health problems due to tracheal stenosis. In the untreated group, four animals were sacrificed for humane reasons before the end of the observation period. In the FIR-SeV/ΔF-treated group, no animal was sacrificed. The FIR-SeV/ΔF-treated animals had a significantly increased survival rate compared to the untreated controls (*P* < 0.05). The Kaplan–Meier survival curves during the five-day follow-up period are shown in Fig. 3. It suggests that FIR-SeV/ΔF improves the survival rate by preventing tracheal stenosis.

### Transgene expression of FIR in tracheal tissues

**Figure 4:**
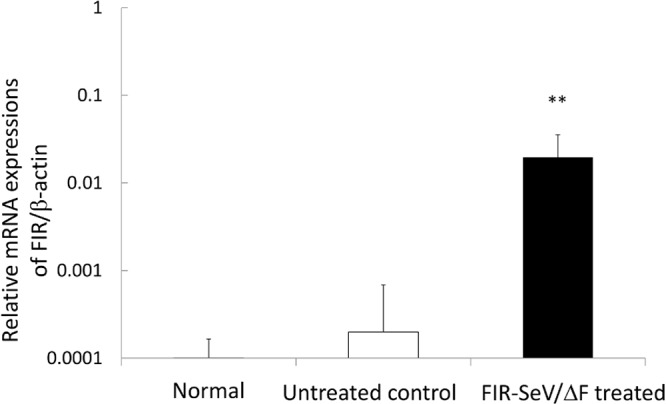
Transgene RNA expression of FIR in the tracheal tissues. To confirm the expression of the FIR transgene in the tracheal tissues, fresh tracheal tissue samples were excised from the normal, the untreated, and the FIR-SeV/ΔF-treated animals (*n* = 3 per group), and were prepared for quantitative real-time RT-PCR analysis. The relative mRNA expression of FIR/β-actin is shown as the mean + SEM (bars) in the three independent experiments. **P < 0.001 using the Mann–Whitney U test. The mRNA expression of FIR was significantly higher in the FIR-SeV/ΔF-treated animals than in the normal and the untreated control animals.

To evaluate the transgene expression of FIR, the mRNA expression of FIR in excised tracheal tissues was determined using quantitative real-time reverse transcription-polymerase chain reaction (qRT-PCR). The relative mRNA expression of FIR was significantly increased in the FIR-SeV/ΔF-treated animals, when compared to the normal or the untreated control animals (1.96 × 10^-2^ ± 1.61 × 10^-2^ vs. 1.01 × 10^-4^ ± 0.64 × 10^-4^ vs. 1.98 × 10^-4^ ± 4.86 × 10^-4^, respectively; *P* < 0.001) (Fig. 4). The expression of FIR following the delivery of the FIR-SeV/ΔF transgene was confirmed in the tracheal tissues.

### c-Myc is suppressed by FIR-SeV/ΔF

**Figure 5:**
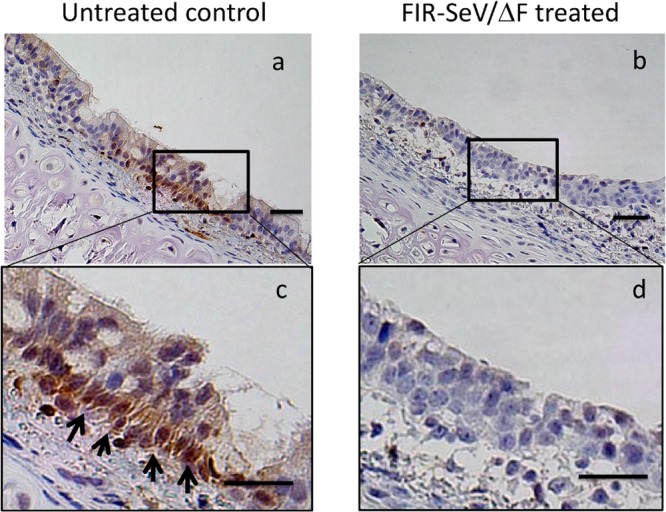
Immunohistochemical analysis of c-Myc expression in the rat trachea. Immunohistochemical staining showed that c-Myc was overexpressed in the tracheal basal cells, which proliferate actively to ensure the renewal of the injured tracheal epithelium (arrows) in the untreated animals (**a, c**), but was downregulated in those same cells in the FIR-SeV/ΔF-treated animals (**b, d**). The scale bar in (**a, b**) and in (**c, d**) indicates 50 μm and 10 μm, respectively.

**Figure 6:**
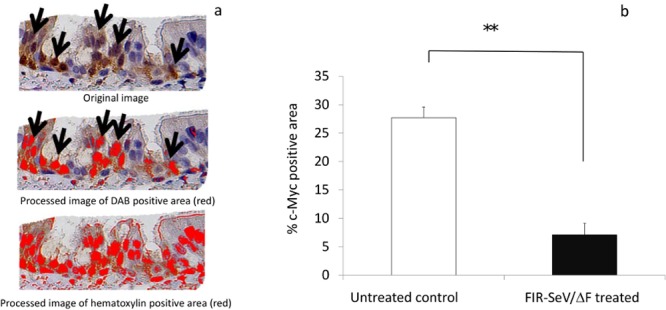
Quantitative analysis of c-Myc expression. To quantify the c-Myc-positive (arrows) area in the nucleus, the percentage of the DAB-stained area in the ROIs, was calculated with a computer imaging software by using the following formula: (DAB-positive area / hematoxylin-positive area) × 100 (**a**). The percentage of the c-Myc-positive area of the tracheal epithelial cells in the FIR-SeV/ΔF-treated group was significantly smaller than that in the untreated controls (**b**). The results are expressed as the mean + SEM (bars). **P < 0.001 using the Mann–Whitney U test.

To evaluate how c-Myc was regulated in the mechanically injured airway mucosa by FIR-SeV/ΔF, immunohistochemical staining using an anti-c-Myc antibody was performed. Representative sections of the paraffin-embedded rat trachea are shown in Fig. 5a–d. c-Myc was mainly overexpressed in the nucleus of the tracheal basal cells of the untreated animals (Fig. 5a,c), but was less expressed in the same cells of the FIR-SeV/ΔF-treated animals (Fig. 5b,d). The area of c-Myc-positive tracheal epithelial cells in the FIR-SeV/ΔF-treated group was significantly smaller than that of the untreated control group (7.09 ± 1.88% vs. 27.7 ± 2.02%, *P* < 0.001) (Fig. 6a,b). c-Myc was probably suppressed by FIR-SeV/ΔF in the regeneration of the airway epithelium of the injured tracheal mucosa.

## Discussion

In the present study, the FIR-SeV/ΔF-treated animals showed a significant decrease of tracheal stenosis and a significant improvement in the survival rate. An elevated expression level of the transgene FIR mRNA was observed in the FIR-SeV/ΔF-treated tracheal tissues. c-Myc was overexpressed in the tracheal basal cells from the area of untreated mucosal injury in the control animals, but was down-regulated in the same cells from the FIR-SeV/ΔF-treated animals. Our data clearly suggests that FIR-SeV/ΔF has therapeutic potential in the treatment of tracheal stenosis through its inhibitory effect on the proliferation of cells in the regenerating airway epithelium.

Owing to the risks of pathological immune responses [22] or of oncogenesis [23] with the current viral vectors, the use of gene therapy is limited to clinical trials for the treatment of cancer or for that of hereditary diseases linked to genetic defects. No experimental study on gene therapy for the treatment of tracheal stenosis in a model of acute mucosal injury has been documented. To make gene therapy for benign airway diseases practical, it will be necessary to develop novel vector systems that efficiently deliver genes to the airway epithelium, but that pose a lower risk of dangerous immune responses or of mutagenesis than the current viral vectors do.

SeV has unique features as a vector, such as a strong affinity for the airway mucosa, a high transduction capacity, an optimal persistence, and sufficient evidence of its therapeutic safety.[6–9] In addition, our previous study revealed the successful transgene expression without any unwanted local immune response, and with an efficient duration when administering SeV/ΔF by spraying to the injured laryngotracheal mucosa.[9] The administration method of spraying is simple and easy; therefore, it shows potential for clinical use in the treatment of human laryngotracheal diseases.

The safety advantage of SeV compared with other viral vectors is that the SeV genome is located exclusively in the cytoplasm of the infected cells, and does not go through a DNA phase; i.e., there is no danger of the undesired integration of foreign sequences into the host chromosomal DNA. Extensive preclinical studies have shown the therapeutic potential of this vector for use in the treatment of airway disease, [6–9] ischemic brain disease, [24] and malignant tumors.[17, 25] In addition, the potential of the SeV vector for vaccination via the airway mucosa by nasal drip administration has been reported.[26, 27] SeV is currently being used in Japan in clinical trials for the treatment of ischemia of the lower limbs, without any reported serious adverse events over a 6-month follow-up.[28] Our SeV vector system has the potential to act as an efficient and safe gene delivery system for the treatment of airway diseases. To our knowledge, this represents the first reported demonstration of successful gene therapy using an SeV vector in a model of acute mucosal injury.

The far upstream element (FUSE)-binding protein (FBP) increases the transcription of *c-myc* by increasing the ability of the transcription factor II human (TFIIH) protein to release the paused RNA polymerase enzyme [15]. This is counteracted by the activity of the FBP-interacting repressor (FIR), which binds to both the FBP and the TFIIH, and down-regulates the helicase activity of TFIIH.[29]

Recent reports indicate the important role of c-Myc not only on tumor growth but also on the wound-healing response.[12] Several preclinical studies have reported the effects of inhibiting c-Myc on the treatment of lung fibrosis, [30] on the stenosis of coronary arteries, [31] and on malignant tumors.[17] c-Myc can be considered an important molecular target to prevent tracheal stenosis due to the unnecessary cell proliferation in the injured airway mucosa. Therefore, the *c-myc* suppressor FIR can be considered a potential candidate for tracheal stenosis therapy.

It is necessary to focus on the time-course of the wound-healing process that occurs in the airway mucosa, when aiming to prevent LTS after a mucosal injury.[32, 33] If the wound-healing response is completed without any unwanted cell proliferation, then the lumen may remain free of stenosis. Immediately following an injury, platelets or thrombocytes aggregate at the injury site to form a fibrin clot during the coagulation phase (i.e., 0–2 days following an injury). Next, the immune cells are activated during the inflammation phase (i.e., 2–3 days following an injury). In the early proliferative phase (i.e., 3–5 days following an injury), the epithelialization usually precedes the activation of cell proliferation in the submucosal layer. The late proliferative phase (i.e., 5–14 days following an injury) is characterized by angiogenesis and fibroblast growth. The remodeling phase (i.e, several weeks following an injury) continues for a prolonged period with the deposition of collagen produced by fibroblasts.[34] Once wound-healing response has advanced to the remodeling phase, surgery is the only treatment option available. However, recurrent stenosis following the surgery has been a long-standing problem. Novel approaches to inhibit cell proliferation in the early phase of wound healing have been needed. In this study, FIR-SeV/ΔF effectively prevented LTS through its suppression of *c-myc* transcription in the proliferative phase, because the airway affinity-based transgene expression of the SeV vector achieved a peak in several days, and then persisted for around two weeks, as we have previously reported.[9]

The tracheal epithelium comprises goblet cells, ciliated cells, and basal cells. The tracheal basal cells represent a multipotent progenitor cell type that proliferates actively to ensure the renewal of the injured tracheal epithelium.[35] In the present study, c-Myc was overexpressed in the tracheal basal cells from the site of untreated mucosal injury in control animals, but was downregulated in the same cells from the FIR-SeV/ΔF-treated animals. The initial thickening of the airway mucosal cells is the process that is mainly responsible for recurrent airway stenosis. The modulation of the wound-healing response following an injury to the airway mucosa is a key component of the treatment of tracheal stenosis. Thus, gene therapy using the *c-myc*-targeting FIR-SeV/ΔF could be an attractive treatment option in the treatment of tracheal stenosis in the future.

The possible concerns of this treatment are the long-term preventive effect and an unwanted delay in the wound-healing process. Once the wound-healing response is completed, it is presumed that the stenosis-preventive effect will be maintained. However, as the observation period in this research project is short, a long-term effect of the transient overexpression of FIR through the use of the recombinant SeV vector should be examined in future experiments. Another concern is that, if the *c-myc* suppression continues for too long, it may impair wound healing undesirably when FIR-SeV/ΔF is used in combination with surgical treatment. The transient expression through SeV/ΔF[9] is considered to be suitable for the limited suppression of the acute phase of the wound-healing response.

Although this research revealed the mechanism of the c-Myc-dependent cell proliferation in the regenerating epithelium, a histological examination of the FIR-treated animals showed that the thickening of the submucosal tissue was also inhibited. An alternative function of FIR, i.e. other than the control of c-Myc, has been suggested and has also been shown in recent research. FIR inhibits the cell-cycle via its disruption of P27Kip1 (P27) expression [36, 37]. P27 arrests cells at the G1 phase, and SAP155, a subunit of the essential splicing factor 3b subcomplex in the spliceosome, is required for proper pre-mRNA splicing by P27. Notably, FIR forms a complex with SAP155, and thus alters the expression of c-Myc and of P27, through the disruption of the well-established functions of FIR and SAP155A.[36, 37] Accordingly, it has been reported that c-Myc is regulated at the transcriptional and post-transcriptional level by the FIR-SAP155 complex.[38] FIR has the potential to inhibit cell proliferation not only by suppressing *c-myc,* but also by blocking the cell cycle via P27. A further investigation of the alternative function of FIR is needed in future experiments.

Considering its enhanced safety, its efficient transgene expression, and its low contact time requirement compared to other vectors that are currently used, our SeV vector can be administered safely and easily to the airway mucosa by inhalation. Therefore, our FIR-encoding SeV vector shows great potential for therapeutic use in a clinical setting, e.g., for prophylactic inhalation for the treatment of the acute phase of an airway burn, for prolonged intubation, and for surgical treatments.

In summary, we have demonstrated that the airway-targeted expression of FIR could efficiently prevent tracheal stenosis, through its suppression of *c-myc* transcription. We believe that the *c-myc*-targeting, SeV-encoding FIR can be developed as a safe and effective therapeutic agent for the treatment of airway disease.
